# Thermodynamic Modeling Study of Carbonation of Portland Cement

**DOI:** 10.3390/ma15145060

**Published:** 2022-07-20

**Authors:** Kamasani Chiranjeevi Reddy, Nahom S. Melaku, Solmoi Park

**Affiliations:** Department of Civil Engineering, Pukyong National University, 45 Yongso-ro, Nam-gu, Busan 48513, Korea; kc.reddy@pknu.ac.kr (K.C.R.); naspknu@pukyong.ac.kr (N.S.M.)

**Keywords:** Portland cement, carbonation, thermodynamic modeling, phase evolution

## Abstract

The assessment of the extent of carbonation and related phase changes is important for the evaluation of the durability aspects of concrete. The phase assemblage of Portland cements with different clinker compositions is evaluated using thermodynamic calculations. Four different compositions of cements, as specified by ASTM cements types I to IV, are considered in this study. Calcite, zeolites, and gypsum were identified as carbonation products. CO_2_ content required for full carbonation had a direct relationship with the initial volume of phases. The CO_2_ required for portlandite determined the initiation of carbonation of C-S-H. A continual decrease in the pH of pore solution and a decrease in Ca/Si is observed with the carbonation of C-S-H. Type II cement exhibited rapid carbonation at relatively less CO_2_for full carbonation, while type III required more CO_2_ to carbonate to the same level as other types of cement. The modeling of carbonation of different Portland cements provided insights into the quantity of CO_2_ required to destabilize different hydrated products into respective carbonated phases.

## 1. Introduction

The interaction of CO_2_ with hydrated cement induces various physiochemical changes in the hydration products and unreacted clinker. Carbon dioxide present in the ambient environment diffuses through the capillary pores of cement and is dissolved in pore solution, resulting in the carbonates at pH ≥ 9 and bicarbonates at 6 ≤ pH ≤ 8 [1,2]. The carbonate ions primarily interact with calcium in hydration products, resulting in the precipitation of calcium carbonate [3]. The formation of carbonates, bicarbonates, and the consumption of calcium during the carbonation reaction results in a decrease in pH [4,5,6]. At these reduced pH levels, the protective oxide layer becomes unstable and exposes reinforcing steel to corrosion [7,8]. Hence, carbonation is one of the most important durability properties to accurately evaluate.

The carbonation of concrete can happen at a wide variety of exposures. Natural carbonation that occurs at atmospheric pressure (PCO_2_—3.55 × 10^−4^ bars) is a very slow process and can progress for many years. Carbonation at higher partial pressures of CO_2_ can accelerate the carbonation rate and help our understanding of the phase change phenomenon over a short period [9]. However, accelerated carbonation can be carried out at various CO_2_ increments by the mass of the binder, and is related to natural carbonation [3]. Alternatively, mathematical models, kinetic models, and thermodynamic models can be employed to quickly predict the phase assemblage resulting from the carbonation of Portland cement [10,11,12].

Thermodynamic calculations and modelling were shown to reliably predict the hydrate assemblage upon complete hydration, changes in solid phase composition and pore solution composition in cementitious systems [13,14]. Numerous studies have employed thermodynamic modelling in assessing phase changes in Portland cement systems with mineral additions such as limestone, clay, fly ash, and slag [15,16,17,18,19,20,21]. Thermodynamic simulations were also applied to predict the hydrate assemblage in alternate cements such as calcium sulfoaluminate cements [22,23,24,25,26] and alkali-activated slag systems [27,28,29]. In addition, thermodynamic calculations are very convenient for assessing the durability performance of cementitious systems. They have been extensively used in predicting phase changes as a function of chloride content using NaCl and sea water in PC systems [30,31] and as a function of CO_2_ in hydrated PC and PC blends subjected to carbonation [32,33].

The extent of the carbonation of cement mainly depends on the diffusion capacity of CO_2_, which in turn depends on the porosity and pore size distribution [4,9]. The carbonation of plain PC primarily happens in portlandite, resulting in CaCO_3_ precipitation and a subsequent decrease in pore size, thereby negating accelerated carbonation in PC [33,34,35]. Portlandite buffers the pore solution pH by supplying [OH^-^] ions and the amount of portlandite determines the onset of carbonation of C-S-H [32,36]. The stability of different phases of hydrated cement depends on the pore solution pH and is destabilized at various pH concentrations when the pH drops below the corresponding threshold value [37]. Thus, the relative proportions of hydrated phases present in hydrated cement tend to influence the onset and extent of carbonation. Hence, it is essential to study the carbonation behavior of hydrated cement with different clinker compositions.

In this study, the carbonation behavior and related changes in phase assemblage of ASTM cement types I-IV were studied using thermodynamic modeling. ASTM types of cement differ in clinker composition, and thus result in hydration products with different proportions, thereby altering the extent of carbonation.

## 2. Materials and Methods

Four types of Portland cement were used in the simulation of the carbonation of Portland cement with a water to cement ratio of 0.8. Up to 40% CO_2_ was added by the mass of cement. A high water-to-cement ratio was used, as it was the minimum ratio that satisfied the mass balance requirement of the whole mixture for the given CO_2_ quantity. The composition of each type of cement is indicated in Table 1. The reaction degree of the clinkers after 28 days of hydration was predicted using Parrot and Killoh’s hydration model and used in the carbonation simulation.

Thermodynamic calculations were made using GEM-Selektor v.3.7 [38,39] and CEMDATA18 [40]. The activity coefficients were calculated using the following equation [41]:

(1) $$log_{10}\gamma_{i}=\frac{-A_{\gamma }z_{i}^{2}\sqrt{I}}{1+\dot{a}B_{\gamma }\sqrt{I}}+b_{\gamma }I+log_{10}\frac{X_{jw}}{X_{w}}$$

where $\gamma_{i}$ and $z_{i}$ represent the activity coefficient and charge of the $i^{th}$ species, respectively. $A_{\gamma }$ and $B_{\gamma }$ are coefficients dependent on temperature and pressure, respectively. The effective molal ionic strength is given by I. $X_{jw}$ and $X_{w}$ are the molar quantity of water and total aqueous phase, respectively. Common ion size parameter $\dot{\left(a\right)}$ and short-range interaction parameter $\left(b_{\gamma }\right)$, were set to 3.67 Å and 1.23 kg/mol, respectively, using KOH as the background electrolyte.

## 3. Results

**Figure 1:**
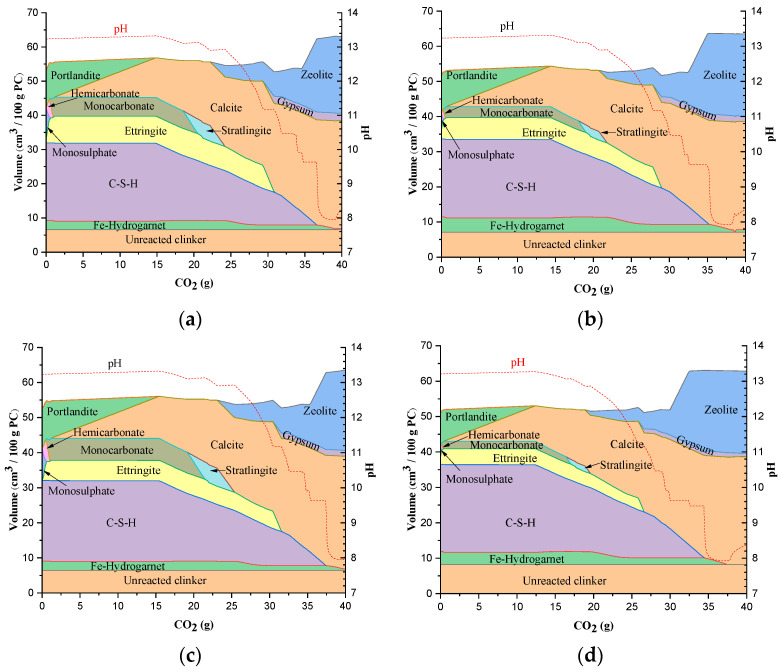
Simulation of carbonation of Portland cement: (**a**) type I, (**b**) type II, (**c**) type III and (**d**) type IV.

The predicted phase assemblages of Portland cement during carbonation are shown in Figure 1 as a function of carbonation extent. C-S-H and portlandite are the major hydration products that are present along with unreacted clinker. Fe-hydrogarnet, monosulphate, and ettringite were the minor phases. The modeling results for the carbonation of all types of Portland cement predict the formation of calcite, zeolites, and gypsum as carbonation products [42,43]. While calcite started forming at a lower carbonation extent, the gypsum and zeolites precipitated at a higher carbonation extent. Strätlingite formed as a transient phase at a higher carbonation extent. The extent of carbonation was observed to depend on the type of cement.

The phase volume change in type I cement as a function of carbonation extent is shown in Figure 1a. Portlandite and monosulphate started to destabilize upon exposure to very little CO_2_, resulting in the formation of hemicarbonate and ettringite. Hemicarbonate further destabilized into monocarbonate, a more stable phase upon the complete destabilization of monosulphate, with an increase in the carbonation extent. The destabilization of portlandite continued until its depletion at an increased carbonation extent. Calcite is the prominent carbonate phase resulting from the destabilization of portlandite; however, the calcite precipitation is initiated upon the complete destabilization of hemicarbonate to monocarbonate. The volume of ettringite increased during monosulphate destabilization and remained constant up to carbonation extent of ~30 g per 100 g cement and then destabilized into gypsum. The kinetics of ettringite destabilization can be expressed with the following equation [44,45]:

(2) $$\mathrm{Ettringite}+\mathrm{Carbon}\;\mathrm{dioxide}\to \mathrm{Calcium}\;\mathrm{carbonate}+\mathrm{gypsum}+\mathrm{alumina}\;\mathrm{gel}+\;\mathrm{water}\;\left(3\mathrm{CaO}\cdot {\mathrm{Al}}_{2}O_{3}\cdot 3{\mathrm{CaSO}}_{4}\cdot 32H_{2}O+3{\mathrm{CO}}_{2}\;\to 3{\mathrm{CaCO}}_{3}+3\left({\mathrm{CaSO}}_{4}\cdot 2H_{2}O\right)+{\mathrm{Al}}_{2}O_{3}\cdot {\mathrm{xH}}_{2}O+\left(26-x\right)H_{2}O\right)$$

The destabilization of C-S-H is triggered with the complete depletion of portlandite and continues until its completion. Calcite is the carbonate phase resulting from the C-S-H destabilization. The pH of the pore solution also started to decline during the carbonation of C-S-H [46]. With a small decrease in pH from 13.3 to 13.1, the monocarbonate is destabilized into strätlingite. Strätlingite is a gehlenite hydrate with a typical composition as Ca_2_Al_2_SiO_7_.8H_2_O and is precipitated from the alumina and silica released by the monocarbonate and C-S-H decomposition, respectively. While the pH is constant during strätlingite formation, it further decreased and reached a small plateau during the destabilization of strätlingite and simultaneous precipitation of zeolites at a pH of ~12.9. Thereafter, the pH rapidly decreased to a value of ~11.2 and again reached a plateau where the ettringite destabilization was initiated. The destabilization of ettringite resulted in the formation of gypsum along with calcite. The volume of zeolites increased close to the complete destabilization of C-S-H. The pH is constant during the precipitation of zeolites; however, it decreased drastically upon the complete consumption of C-S-H. The formation of zeolites is mainly attributed to the destabilization of strätlingite and C-S-H coupled with the availability of alkali metals in the pore solution. The formation of zeolites reduces the alkali metal ions in the pore solution, resulting in the decrease in pH [32]. Fe-hydrogarnet partially destabilized into calcite after strätlingite consumption, while its complete destabilization occurred after the total carbonation of C-S-H.

The volume changes in different phases in hydrated type II cement when exposed to CO_2_ are shown in Figure 1b. Type II cement has a lower amount of hydration products with the exception of Fe-hydrogarnet and ettringite compared to type I cement. Portlandite and monosulphate begin to carbonate immediately, as noticed in the previous case. Monosulphate is completely destabilized to ettringite and hemicarbonate. At a similar extent of carbonation with that of type I cement, monocarbonate started to precipitate. A small decrease in the hydrogarnet volume is observed during decomposition of hemicarbonate.

While the relative trends in carbonation of different phases are similar to the type I cement, the initial volume of phases determined the amount of CO_2_ required for the full carbonation. The complete carbonation of portlandite in type II occurred at a relatively lower extent of carbonation when compared to type I (i.e., 14.6 g CO_2_). This initiated the early destabilization of C-S-H and a decrease in the pH value in the system from 13.3. The destabilization of monocarbonate to strätlingite, and further destabilization of strätlingite to calcite and zeolites were also advanced. Ettringite destabilization is also initiated at a lower carbonation extent. The complete carbonation of C-S-H to calcite in type II cement is also advanced, thus causing the destabilization of Fe-hydrogarnet. The changes in pH in type II cement followed a similar pattern to that of type I cement; however, the decrease in pH occurred at a lower carbonation extent compared to type I cement, owing to the lower quantity of hydration products.

Figure 1c shows the changes in the volume of different phases in type III cement in the CO_2_ environment. Type III cement has the highest amount of hydration products compared to the other cement types. As a result, the CO_2_ content required to destabilize the phases into their respective stable phases was higher compared to the other types of cement. Ettringite, unlike in other types of cements, was not a hydration product but precipitated through destabilization of monosulphate to monocarbonate via hemicarbonate. The destabilization of phases at various levels of CO_2_ and changes in the pore solution pH follow a similar trend to that of type I and type II cements. The last type of cement, type IV, has the lowest amount of hydration products with the exception of C-S-H. It has a highest amount of C-S-H which can be attributed to the higher amount of C_2_S. The volume changes in phases of type IV cement during carbonation are shown in Figure 1d. The lesser volume of hydration phases resulted in the carbonation of phases at lower contents of CO_2_ than the other types of cement.

**Figure 2:**
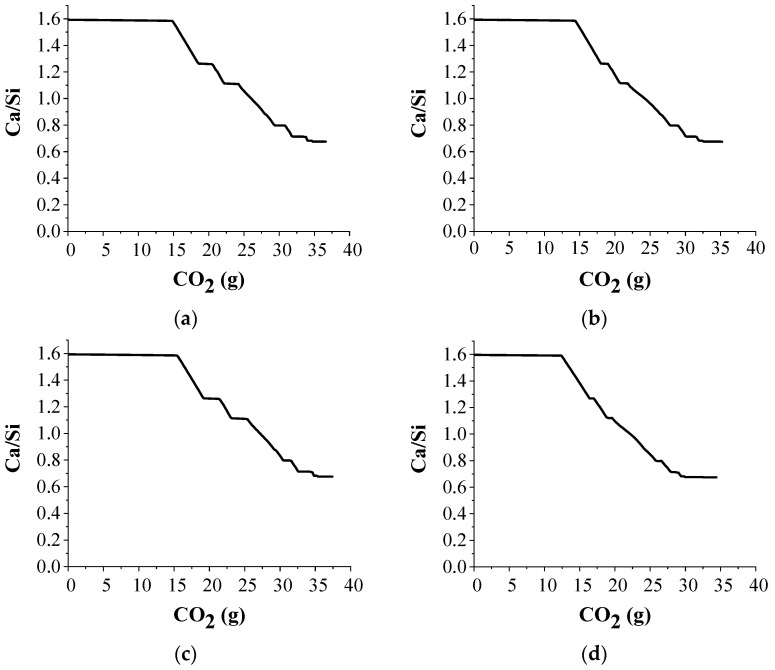
Change in the Ca/Si ratio of C-S-H during carbonation. (**a**) type I, (**b**) type II, (**c**) type III, (**d**) type IV.

The change in the Ca/Si ratio of C-S-H during the simulated carbonation is shown in Figure 2. The volume of C-S-H is constant during the carbonation of portlandite, as the portlandite preferentially undergoes carbonation and acts as a buffer for C-S-H [47]. Therefore, the Ca/Si ratio of C-S-H is constant as long as portlandite is present in the system. Upon the consumption of portlandite, C-S-H starts destabilizing into calcite resulting in Si-rich C-S-H gel with a continuous decrease in the Ca/Si ratio, until the destabilization of monocarbonate is initiated. During the destabilization of monocarbonate to strätlingite, the Ca/Si ratio is constant. The precipitation of strätlingite consumes the Si released by the carbonation of C-S-H, thereby offsetting the decrease in the Ca/Si ratio of C-S-H. The Ca/Si ratio of C-S-H continues to decrease upon the complete stabilization of strätlingite until the destabilization of strätlingite is initiated. The Si released from the combined carbonation of C-S-H and destabilization of strätlingite along with Al from strätlingite is used in the stabilization of zeolites, and this ensures a constant Ca/Si during the destabilization of strätlingite. The Ca/Si ratio continues to decrease after the complete carbonation of strätlingite till the conversion of ettringite into gypsum is initiated. During the destabilization of ettringite, the Al released from ettringite and Si from C-S-H again results in zeolites, leading to a constant Ca/Si ratio of C-S-H. The Ca/Si further decreases post ettringite carbonation and reaches a plateau when C-S-H quantity becomes very low. The increase in the volume of zeolites is observed during the constant Ca/Si ratio of C-S-H prior to the complete carbonation of C-S-H.

## 4. Conclusions

This study investigated the effect of the chemical compositions of cements on the phase evolution during carbonation using thermodynamic calculations. The outcomes of this study can be summarized as follows.

(1) Calcite is the major carbonation product. Zeolites and gypsum were observed as other minor carbonation phases, while the carbonation of the major hydration products resulted in calcite and the destabilization of strätlingite and C-S-H, along with the alkali metal ions present in the pore solution, resulted in zeolites. On the other hand, the formation of gypsum is solely linked to the destabilization of ettringite.
(2) The initial volume of each phase determined the CO_2_ content required for their full carbonation. The higher the initial volume, the larger the CO_2_ content required for full carbonation.
(3) The extent of CO_2_ required for the initiation of C-S-H carbonation has a direct relationship with portlandite quantity. Pore solution pH and Ca/Si of C-S-H continually decreased with the carbonation of C-S-H.
(4) The carbonation of type II cement is rapid and progressed at a relatively lower CO_2_ content, while type III had a higher demand of CO_2_ for a similar extent of carbonation compared to other types of cements. The volume of hydration products prior to carbonation determined the quantity of CO_2_ required for complete carbonation.
(5) Type IV cement required a greater content of CO_2_ for complete carbonation of C-S-H owing to the highest content of C-S-H.

The results reveal that a higher portlandite content delays the carbonation of C-S-H and the quantity of C-S-H itself determines the amount of CO2 required for its complete destabilization. The clinker composition, which can result in large quantities of portland-ite and C-S-H can be most effective under carbonation conditions. With further studies, the type of cement with the best mechanical CO2 resistance and other durability perfor-mances can be recommended. Further experimental programs can be designed to include compressive strength tests, XRD and TGA profiling, and other microstructure evaluation techniques on carbonated PC and its blends with other cementitious materials for a de-tailed understanding of the carbonation mechanism and its effect on strength and dura-bility. 

## Figures and Tables

**Table 1:** Composition of PC (expressed in mass %).

	C_3_S	C_2_S	C_3_A	C_4_AF	CaSO_4_	Other Phases
Type I	55	19	10	7	4.2	4.8
Type II	51	24	6	11	3.3	4.7
Type III	57	17.7	10	7	3.1	5.2
Type IV	38	43	4	9	2.4	3.6

## Data Availability

The data are available upon request.
